# High signal-to-noise imaging of spontaneous and 5 ns electric pulse-evoked Ca^2+^ signals in GCaMP6f-expressing adrenal chromaffin cells isolated from transgenic mice

**DOI:** 10.1371/journal.pone.0283736

**Published:** 2023-03-31

**Authors:** Ciara Viola, Thomas W. Gould, Nicole Procacci, Normand Leblanc, Josette Zaklit, Gale L. Craviso

**Affiliations:** 1 Department of Pharmacology, University of Nevada Reno School of Medicine, Reno, Nevada, United States of America; 2 Department of Physiology and Cell Biology, University of Nevada Reno School of Medicine, Reno, Nevada, United States of America; 3 Department of Electrical and Biomedical Engineering, College of Engineering, University of Nevada Reno, Reno, Nevada, United States of America; Cinvestav-IPN, MEXICO

## Abstract

In studies exploring the potential for nanosecond duration electric pulses to serve as a novel modality for neuromodulation, we found that a 5 ns pulse triggers an immediate rise in [Ca^2+^]_i_ in isolated bovine adrenal chromaffin cells. To facilitate ongoing efforts to understand underlying mechanisms and to work toward carrying out investigations in cells *in situ*, we describe the suitability and advantages of using isolated murine adrenal chromaffin cells expressing, in a Cre-dependent manner, the genetically-encoded Ca^2+^indicator GCaMP6f. Initial experiments confirmed that Ca^2+^ responses evoked by a 5 ns pulse were similar between fluorescent Ca^2+^ indicator-loaded murine and bovine chromaffin cells, thereby establishing that 5 ns-elicited excitation of chromaffin cells occurs reproducibly across species. In GCaMP6f-expressing murine chromaffin cells, spontaneous Ca^2+^ activity as well as nicotinic receptor agonist- and 5 ns evoked-Ca^2+^ responses consistently displayed similar kinetic characteristics as those in dye-loaded cells but with two-twentyfold greater amplitudes and without photobleaching. The high signal-to-noise ratio of evoked Ca^2+^ responses as well as spontaneous Ca^2+^ activity was observed in cells derived from *Sox10-Cre*, conditional GCaMP6f mice or *TH-Cre*, conditional GCaMP6f mice, although the number of cells expressing GCaMP6f at sufficiently high levels for achieving high signal-to-noise ratios was greater in *Sox10-Cre* mice. As in bovine cells, Ca^2+^ responses elicited in murine GCaMP6f-expressing cells by a 5 ns pulse were mediated by the activation of voltage-gated Ca^2+^ channels but not tetrodotoxin-sensitive voltage-gated Na^+^ channels. We conclude that genetically targeting GCaMP6f expression to murine chromaffin cells represents a sensitive and valuable approach to investigate spontaneous, receptor agonist- and nanosecond electric pulse-induced Ca^2+^ responses *in vitro*. This approach will also facilitate future studies investigating the effects of ultrashort electric pulses on cells in *ex vivo* slices of adrenal gland, which will lay the foundation for using nanosecond electric pulses to stimulate neurosecretion *in vivo*.

## Introduction

Electrical stimulation approaches that target the central or peripheral nervous system are becoming more widespread for treating neurological, psychiatric and visceral disorders. Current methods for delivering an electric stimulus range from invasive surgical implantation of electrodes [1–3] to noninvasive strategies in which electrodes are positioned externally on the scalp [4–6] or on the skin [3,7] for brain and peripheral nerve stimulation, respectively. Given the overall success of this form of neuromodulation, novel electrostimulation approaches are being sought that not only are safe and effective with few adverse side effects but also can offer advantages over existing methods.

A promising new approach for neuromodulation is the use of electric pulses that are nanosecond in duration [8,9]. As opposed to electric stimulation protocols currently used in the clinical setting, which rely on milli- and microsecond duration electric pulses, pulse durations in the nanosecond range have the potential to be delivered remotely to achieve deep-penetration and precise targeting of nerves and excitable tissues within the body including brain [10–13]. Moreover, these ultrashort pulses also present novel and unique ways to fine tune neural cell excitability by varying the pulse duration [14,15], the pulse waveform (i.e., including a reverse polarity phase) [15–18] and the delivery method (i.e., single pulse versus a pulse pair [19]; a single threshold pulse versus high repetition rate bursts of subthreshold pulses [20,21]). Importantly, when used as a stimulus, nanosecond electric pulses (NEP) have been shown to elicit damage-free excitation of isolated rat nociceptor neurons [22] and cultured mouse hippocampal neurons [23]. When delivered as high frequency trains, electrostimulation by NEP can elicit action potentials in *ex vivo* preparations of the frog sciatic nerve [15,18,20,24] without causing fatigue or damage to the nerve fibers. Work from our group has shown that single NEP less than 10 ns in duration induce a rise of [Ca^2+^]_i_ in neuroendocrine bovine adrenal chromaffin cells (ACC) that is mediated by Ca^2+^ influx solely through voltage-gated Ca^2+^ channels (VGCC) [17,25–27], mimicking the molecular mechanism by which the physiological activation of these cells occurs *in vivo* by acetylcholine, which is released from the innervating splanchnic nerve and stimulates ACC nicotinic cholinergic receptors [28,29]. Notably, the NEP-evoked rise in [Ca^2+^]_i_ has a functional consequence, the release of catecholamines [26,30]. Based on these results, we have continued to use isolated ACC as a neural-type cell model for elucidating the mechanisms by which NEP modulate cell excitability and neurosecretion.

ACC are embryonically derived from the neural crest and share many similarities to sympathetic neurons that include the synthesis, storage and secretion of catecholamines, the latter occurring by exocytosis, the same Ca^2+^-dependent mechanism used by neurons to release neurotransmitters. These cells play a crucial role in maintaining homeostasis as well as mediating the “fight” or “flight” response to acute stressors. When isolated from adrenal medullary tissue and placed in culture, ACC have long been considered a leading, non-transformed model of neurosecretory/neural-type cells [31], with ACC obtained from bovine adrenal glands being the most extensively characterized due to the large size of the glands and hence the large quantity of cells that can be obtained. In an effort to further elucidate the mechanism underlying ACC excitation by ultrashort NEP, we recently focused on developing a genetic strategy for monitoring [Ca^2+^]_i_ in ACC which, unlike our current approach in which cells are loaded with a fluorescent Ca^2+^ indicator dye, would provide a high signal-to-noise ratio (SNR) to image Ca^2+^ responses.

The ability to achieve enhanced imaging of Ca^2+^ responses has been greatly facilitated by the use of genetically-encoded Ca^2+^ indicators (GECI) that like Ca^2+^ indicator dyes such as Fluo-4 and Calcium Green-1 [32], display a marked increase in fluorescence upon Ca^2+^ binding [33,34]. The most commonly used GECI in neuroscience research are the GCaMP family of proteins, which consist of a circular permutated green fluorescent protein (cpGFP) fused with a calmodulin Ca^2+^-sensing domain [35–37]. GECIs can be expressed in cells *in vitro* by transient transfection, an approach used by Carr et al. [38] to demonstrate the advantages of using GCaMPs for investigating responses to NEP in human glioblastoma cells. However, our attempts to transfect bovine ACC have resulted in low transfection efficiencies, differences in the level of expression between cells, morphological anomalies, and impaired cell viability. Moreover, transfection is less suitable for targeting GECI expression to ACC in a multicellular tissue, such as the adrenal medulla, to image spontaneous and stimulus-evoked Ca^2+^ events, a goal of future studies. Given these limitations, we opted instead to generate transgenic mice conditionally expressing a GECI in ACC. The approach would result in robust expression of Cre recombinase-dependent transgenes in cells within a genetically defined subtype, in this case ACC, and thus provide the opportunity to evaluate NEP-induced Ca^2+^ responses in ACC both *in vitro* and in acutely prepared *ex vivo* slices of the adrenal gland. Because our previous work on the effects of NEP has been performed exclusively in bovine ACC [17,25–27,30], the first goal of this study was to compare NEP-elicited ACC Ca^2+^ responses between dye-loaded bovine and murine cells. The second goal was to validate this new murine transgenic model by determining whether ACC isolated from wild-type (*wt*) mice and from mice expressing a GECI respond similarly to both NEP stimulation and to nicotinic acetylcholine receptor (nAChR) stimulation.

We chose GCaMP6f as the GECI to be expressed due to its fast kinetics and reports of a high SNR (up to ~ 3,000-fold change in fluorescence in some cases) of its florescence signal [33,39]. Two transgenic mouse lines expressing Cre recombinase from different promoters likely to be active in ACC (i.e., Cre-drivers) were selected to target GCaMP6f expression to ACC. Our main findings were that bovine and *wt* murine ACC loaded with a Ca^2+^ indicator dye each undergo an immediate rise in [Ca^2+^]_i_ in response to a 5 ns pulse. Similar to dye-loaded mouse ACC, GCaMP6f-expressing mouse ACC also exhibited an immediate rise in Ca^2+^ in response to NEP stimulation or to nicotinic receptor stimulation. However, stimulus-evoked Ca^2+^ responses in GECI-expressing mouse ACC were of significantly larger amplitudes than those of dye-loaded ACC. Spontaneous Ca^2+^ events were also higher in amplitude in GECI-expressing mouse ACC, and there was no evidence of adverse cellular effects. Finally, Cre-driving mice were found to target the expression of Cre-dependent transgenes to ACC with different selectivity and recombination efficiency. Taken as a whole, these results highlight the suitability of *in vivo* targeting of GCaMP6f expression to mouse ACC for studying Ca^2+^ responses evoked by NEP and other stimuli, as well as for investigating mechanisms underlying spontaneous Ca^2+^ events.

## Materials and methods

### Transgenic mouse generation

Animal studies were performed in accordance with the National Institutes of Health *Guide for the Care and Use of Laboratory Animals* and with animal protocols approved in writing by the Institutional Animal Care and Use Committee at the University of Nevada, Reno. All mice were obtained from The Jackson Laboratory (Bar Harbor, ME). Transgenic mouse lines consisted of mice expressing one copy of conditional, Cre-dependent GCaMP6f (Jax# 24105) as well as Cre recombinase under the control of the rat tyrosine hydroxylase (TH) promoter (Jax# 008601; [40]) or under control of the mouse Sox10 promoter (Jax# 25807). Expression of GCaMP6f in TH-Cre, conditional GCaMP6f mice (heretofore referred to as *TH-GCaMP6f* mice) and Sox10-Cre, conditional GCaMP6 mice (heretofore referred to as *Sox10-GCaMP6f* mice), was maintained in ACC into adulthood as expected, based on the design of the Rosa26 lox-stop-lox targeting constructs in which Cre-mediated excision of the floxed stop cassette that lies upstream of the reporter (in this case GCAMP6f) results in the expression of the reporter under the strong CAG promoter selectively in Cre-positive cells. For this study, adrenal glands were obtained from male and female *wt* mice and transgenic mice ranging in age from 6 to 8 weeks. The genotype of the transgenic mice was confirmed by PCR of genomic DNA isolated from tails or ears. The background strain of all mice used in this study was C57BL/6J.

### Cell isolation, culturing and preparation

The procedure that was used to prepare cultured ACC was modified from Domínguez et al. [41]. Briefly, *wt*, *Sox10-GCaMP6f* or *TH-GCaMP6f* mice were anesthetized by isoflurane inhalation and sacrificed by cervical dislocation. An abdominal incision was made to provide access to both adrenal glands, which were removed and placed in ice-cold Hanks Ca^2+^/Mg^2+^ free balanced salt solution (HBSS) consisting of 145 mM NaCl, 5 mM KCl, 1.2 mM NaH_2_PO_4_, 10 mM glucose, and 15 mM HEPES, pH 7.4. Fat was removed from the glands and the medulla obtained by carefully dissecting away the outer cortex. The medulla from both glands were incubated together for 45 min at 37°C in 220 μl HBSS containing 18–20 U/ml papain, 1 mg/ml deoxyribonuclease (DNAse), 1 mM dithiothreitol (DTT), and 0.2% bovine serum albumin (BSA). The digested tissue was triturated through a series of plastic pipette tips of decreasing diameter until single cells were obtained, which were resuspended in 1:1 DMEM/F-12 medium supplemented with 10% fetal calf serum, 100 U/ml penicillin, 100 μg/ml streptomycin and 0.25 μg/ml fungizone. A 100–150 μl aliquot of the cells was placed in the center of a 35 mm glass-bottom culture dish coated with either collagen or poly-d-lysine. The culture dishes were placed in a tissue culture incubator for 60 min to allow the cells to settle and to attach to the cell dish surface. Then, 2 ml of supplemented DMEM/F-12 medium was gently added to each dish and the cells returned to the cell culture incubator that was maintained at 36.5°C under a humidified atmosphere of 5% CO_2_. The cells were used within 1–2 days of plating. Typically, 6–9 dishes of cells were obtained from both medullae of a single mouse.

### Ca^2+^ imaging

For fluorescence imaging of [Ca^2+^]_i_ in *wt* ACC, cells were incubated with 1 μM of the fluorescent dye Calcium Green-1 AM (480Ex/535Em nm) for 45 min at 37°C in a balanced salt solution (BSS) with the following composition: 145 mM NaCl, 5 mM KCl, 1.2 mM NaH_2_PO_4_, 1.3 mM MgCl_2_, 2 mM CaCl_2_, 10 mM glucose, 0.1% BSA, 15 mM Hepes, pH 7.4. After incubation, the BSS containing the Ca^2+^ indicator was removed and replaced with dye-free BSS lacking BSA. The dish of cells was placed on the stage of a Nikon TE 2000 epifluorescence microscope. Cells were viewed with a 100X air objective and brightfield and fluorescence images were captured with an iXonEM + DU-897 EMCCD camera (Andor, Oxford Instruments, Belfast, Northern Ireland) and open-source Micro-Manager software versions 1.4 and 2.0 gamma. The exposure time of the camera was set to 100 ms and fluorescence images captured at a rate of 7 frames/s. Baseline Ca^2+^ fluorescence of the cells was monitored 10 s prior to stimulus application and continued for 50 s afterwards. For fluorescence imaging of [Ca^2+^]_i_ in ACC expressing GCaMP6f (496_Ex_/513_Em_ nm), the culture medium was removed, replaced with BSS and the dish of cells placed on the microscope stage, with no other manipulations prior to imaging. All experiments were performed at ambient room temperature. When a stimulus was applied, the change in fluorescence intensity of the cells was calculated by subtracting the cell-free background fluorescence from the fluorescence of the cell (F = F_*cell*_−F_*background*_). F was then normalized to the fluorescence intensity value (F_0_) at the time of stimulus delivery (F/F_0_). All fluorescence responses were within the dynamic range of the camera sensor since histograms of pixel intensities monitored during spontaneous and stimulus-evoked fluorescence changes failed to show activation of pixels residing at the highest intensity value of the camera.

For experiments conducted both in the presence and absence of extracellular Ca^2+^, a coverslip containing fibronectin-attached cells was placed inside a perfusion chamber that was positioned on the stage of the microscope. Cells were perfused at a rate of 1 ml/min at ambient room temperature first with BSS containing 2 mM Ca^2+^, then with Ca^2+^-free BSS containing 1 mM EGTA. For these experiments, cells were viewed with a 60X air objective and brightfield and fluorescence images captured as described above.

### Nanoelectropulse delivery

The system used for delivering 5 ns electric pulses to murine ACC was the same as that used in our previous studies that employed bovine ACC [42,43]. Briefly, a single pulse was applied to a cell by means of two cylindrical gold-coated tungsten rod electrodes (diameter of rods 127 μm) in which the electrode tips were spaced 100 μm apart. The electrodes were immersed in the BSS and positioned 40 μm above the bottom of the dish by a motorized MP-225 micromanipulator (Sutter Instruments, Novato, CA), with the cell being exposed to the pulse positioned in the center of the gap between the electrode tips. Pulses 5 ns in duration [42,43] were generated by a custom-fabricated pulse generator (Transient Plasma Systems, Inc., Torrance CA) and delivered to the electrodes at amplitudes that produced an electric field of 8 MV/m at the location of the cell. Delivery of pulses was triggered externally by a program written in LabVIEW and each pulse trace was captured with an oscilloscope. The electric field distribution in the vicinity and at the location of the target cell was computed using the Finite-Difference Time-Domain software package SEMCAD X (version 14.8.5, SPEAG, Zurich, Switzerland) as previously described [42,43].

### Physiological stimulus delivery

The nAChR agonist 1,1-dimethyl-4-piperazinium (DMPP) was applied to an individual cell at a concentration of 50 μM in BSS for a duration of 5 ms using a Picospritzer pressure ejection system (Parker Hannifin, Hollis NH) as described previously [42]. Glass micropipettes used for agonist delivery were placed at a distance of 1.5-cell diameters away from the target cell using a MP-225 motorized micromanipulator (Sutter Instruments, Novato, CA).

### Immunocytochemistry

After 1–2 days in culture, except where noted, *wt* and GCaMP6f-expressing murine ACC were rinsed with phosphate-buffered saline (PBS) and fixed in 4% paraformaldehyde in PBS for 10 min. Cultures were blocked in PBS containing 0.1% Triton X-100 (PBST) and 10% fetal bovine serum (FBS) and incubated overnight at 4°C with antibodies against TH (rabbit, PA5-85167, ThermoFisher, Waltham, MA, USA), phenylethanolamine N-methyltransferase (PNMT) (rabbit, AB110, MilliporeSigma, Burlington, MA, USA), or S100β (rabbit, GA50461-2, Agilent, Santa Clara, CA, USA). For GCaMP6f-expressing cells, the overnight incubation included a green fluorescent protein (GFP) antibody (goat, 600-101-215, Rockland Immunochemicals, Pottstown, PA, USA). Primary antibodies were diluted 1:1000. Cells were then rinsed several times with PBS and incubated for one hour at room temperature in the dark with AlexaFluor 594-conjugated donkey-anti-rabbit and/or AlexaFluor 488-conjugated donkey anti-goat antibodies (ThermoFisher). Secondary antibodies were diluted 1:500 in 10% FBS in PBST that also contained 1 μg/ml bisbenzimide (Hoechst 33342; ThermoFisher) to label nuclei. Cells were rinsed with PBS and brightfield and fluorescence images were acquired using a Nikon TE 2000 epifluorescence microscope equipped with an iXonEM + DU-897 EMCCD camera (Andor).

### Statistical analysis

Each series of experiments was repeated two or more times using ACC obtained from male and female mice from different litters. Responses from each sex were similar and therefore pooled. For all experiments, the number of cells is reported as *c* and the number of mice is reported as *n*. The normalized Ca^2+^ responses of the cells are represented as the mean ± standard deviation (SD) in individual experiments and as the mean ± standard error of the mean (SEM) when averaged between 2 or more experiments. An unpaired Student’s *t* test was used to determine the statistical significance between two means. *p*-values < 0.05 were considered statistically significant.

### Reagents

DMEM/F-12, antibiotics and antimycotics were obtained from Gibco Laboratories (Grand Island, NY, USA), papain and DNase from Worthington Biochemical Corporation (Lakewood, NJ, USA) and FBS from Lonza (Basel, Switzerland). Calcium Green-1 AM was purchased from Molecular Probes (Eugene, OR, USA), tetrodotoxin from EMD Chemicals (San Diego, CA, USA), and ω-conotoxin, ω-agatoxin and SNX-482 from Alomone Labs (Jerusalem, Israel). DMPP, poly-d-lysine, fibronectin, collagen and nifedipine were obtained from Millipore Sigma (St. Louis, MO, USA). All other chemicals were reagent grade and purchased from standard commercial sources.

## Results and discussion

### Characteristics of ACC cultures from *wt*, *Sox10-GCaMP6f* and *TH-GCaMP6f* mice

#### General features

**Fig 1:**
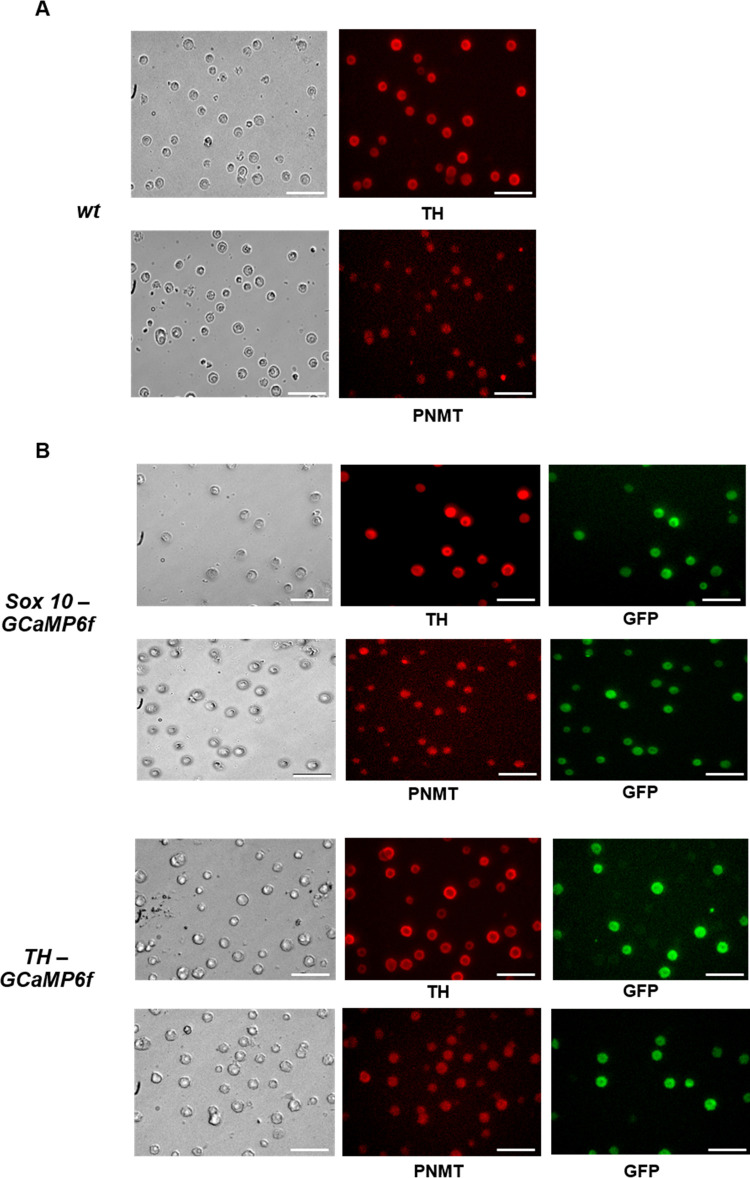
Comparison of cultured cells obtained from the adrenal medulla of *wt*, *Sox10-GCaMP6f* and *TH-GCaMP* mice. (A) Representative brightfield and fluorescence images of *wt* mouse ACC stained with antibodies against TH or PNMT. (B) Representative brightfield and fluorescence images of ACC derived from *Sox10-GCaMP6f* or *TH-GCaMP6f* mice stained with antibodies against TH and GFP, or PNMT and GFP. Scale bar = 100 μm. Hoechst 33342 staining that was used to label nuclei to obtain the total number of cells in each field of view is not shown.

The two transgenic mice used in this study were *Sox10-GCaMP6f* mice that drive GCaMP6f expression in neural crest derivatives, which include ACC, and *TH-GCaMP6f* mice that target GCaMP6f expression to catecholaminergic cells. We first compared the characteristics of cell cultures obtained from the adrenal medulla of *wt*, *Sox10-GCaMP6f* and *TH-GCaMP6f* mice. For each mouse type, an individual culture comprised cells isolated from both adrenal glands of a single mouse. In general, the size and shape of the adrenal glands obtained from *wt* and transgenic mice were similar. Once the outer cortex was removed from the glands, the adrenal medulla from each transgenic mouse had the same healthy appearance as medullary tissue obtained from a *wt* mouse. Following digestion of the medullas, comparable cell yields were obtained from *wt* and transgenic mice. As shown in Fig 1A and 1B, brightfield images of one-day-old cells for each culture were indistinguishable, with no morphological differences apparent at the low magnification used for obtaining the images. Thus, there was no gross evidence of adrenal abnormalities or cell damage induced by GCaMP6f expression.

#### Immunocytochemical analysis

The purity of each culture was assessed next by immunostaining for TH, the rate-limiting enzyme of catecholamine biosynthesis, one to two days after plating. Across *wt* cultures, the percentage of cells that were TH^+^ ranged from 74 to 92% (Table 1), with a similar percentage of cells taking up the dye neutral red that selectively stains monoamine-containing cells [44,45]. For the *wt* culture image shown in Fig 1A, 92% of the cells were TH^+^ positive. In the same culture the predominant catecholamine-producing cell type was determined by immunostaining for PNMT, which catalyzes the conversion of norepinephrine to epinephrine. As shown in Fig 1A, similar to TH, the vast majority of the cells (91%) expressed PNMT (Table 1). Thus, the main catecholamine phenotype of ACC in *wt* cultures was adrenergic, consistent with the higher percentage of adrenergic versus noradrenergic cells (70–80%) reported in ACC across species [46].

**Table 1:** Immunocytochemical characterization of *wt* and transgenic mouse adrenal medullary cultures.

	Total	TH^+^ Cells		GFP^+^ Cells		Total	PNMT^+^ Cells			GFP^+^ Cells	
	(n)^a^	(n)	(%)^b^	(n)	(%)^b^	(n)^a^	(n)	(%)^b^	(n)		(%)^b^
***wt***	132	122	92	N/A	N/A	108	98	91		N/A	N/A
	19	14	74	N/A	N/A	---	---	---		N/A	N/A
***Sox10-GCaMP6f***	97	74	76	74	76	95	78	82		78	82
	35	26	74	26	74	---	---	---		---	---
	16	13	81	13	81	---	---	---		---	---
***TH-GCaMP6f***	128	103	80	47	37	139	117	84		44	32
	200	139	70	15	8	170	126	74		11	6
	313	198	63	198	63	---	---	---		---	---

^a^ total number of cells viewed for an individual culture.

^b^ average of 2 or 3 fields of view per dish.

Immunostaining for TH in cell cultures derived from *Sox-10 GCaMP6f* mice revealed a comparable purity of the cultures in which 76–81% of the cells were TH^+^ (Table 1). As shown in Fig 1B (top panels), all TH^+^ cells co-expressed GFP, a component of GCaMP6f. This phenomenon was highly reproducible in cultures derived from male and female mice from different litters (Table 1). In agreement with this finding, adrenal gland slices from *Sox10-GCaMP6f* mice that were similarly probed with antibodies to TH and GFP showed that GCaMP6f expression was detected in 100% of the targeted ACC population (S1 Fig). Thus, GCaMP6f expression was not lost when ACC from *Sox10-GCaMP6f* mice were isolated and maintained in culture.

ACC derived from *Sox-10 GCaMP6f* mice that were PNMT^+^ were also GFP^+^ (Table 1). The high percentage of cells expressing TH and PNMT suggested that the cultures lacked satellite glial cells (SGC)/sustentacular cells/Schwann cells, which also are derived from the neural crest. These cells are present in the adrenal medulla [47,48] and thus likely dissociated together with ACC. Indeed, cell cultures derived from either *wt* or transgenic mice that were stained with antibodies against S100, a calcium-binding protein highly expressed by peripheral, neural crest-derived glia such as adrenal SGC [49,50], failed to show the presence of S100^+^ cells, which were always detected in adrenal slices (S2 Fig). This result was important since SGC, as noted above, are derived from the neural crest similar to ACC and thus also express Sox10. In other words, SGC would be expected to express GCaMP6f in these cultures if they adhered and survived. Taken together, these results demonstrate that cell cultures derived from the adrenal medulla of *wt* and *Sox10-GCaMP6f* mice are each highly enriched in adrenergic ACC, and that in cultures prepared from *Sox10-GCaMP6f* mice, all ACC co-express GCaMP6f.

Although the purity of adrenal medullary cultures derived from *TH-GCaMP6f* mice was comparable to that from *wt* and *Sox10-GCaMP6f* mice (Table 1), the percentage of TH^+^ cells that co-expressed GFP was more variable, ranging from 8 to 63%. Fig 1B (bottom panels) shows an example of such low co-expression of GFP in TH^+^ cells, which was also the case for expression of GFP in PNMT^+^ cells. Immunocytochemical analysis of freshly isolated cells yielded the same results, indicating that the low percentage of GFP^+^ ACC cells was not caused by the loss of GFP expression in culture. In fact, adrenal gland slices from *TH-GCaMP6f* mice probed with antibodies to TH and GFP revealed that GCaMP6f was not expressed in all TH^+^ cells (S1 Fig), a finding consistent with the original report on the transgenic *TH-GCaMP6f* mouse line used in this study [40] in which images of the adrenal gland, as well as other catecholaminergic structures, showed reporter expression in a similarly mosaic pattern. Thus, for reasons that are unclear, fewer TH^+^ ACC expressed GCaMP6f in adrenal medullary tissue and cell cultures derived from *TH-GCaMP6* mice than in adrenal medullary tissue and cell cultures derived from *Sox10-GCaMP6f* mice.

### Evaluation of morphology as well as basal and spontaneous fluctuations of fluorescence in *wt* dye-loaded cells versus GCaMP6f-expressing cells

#### Morphology

**Fig 2:**
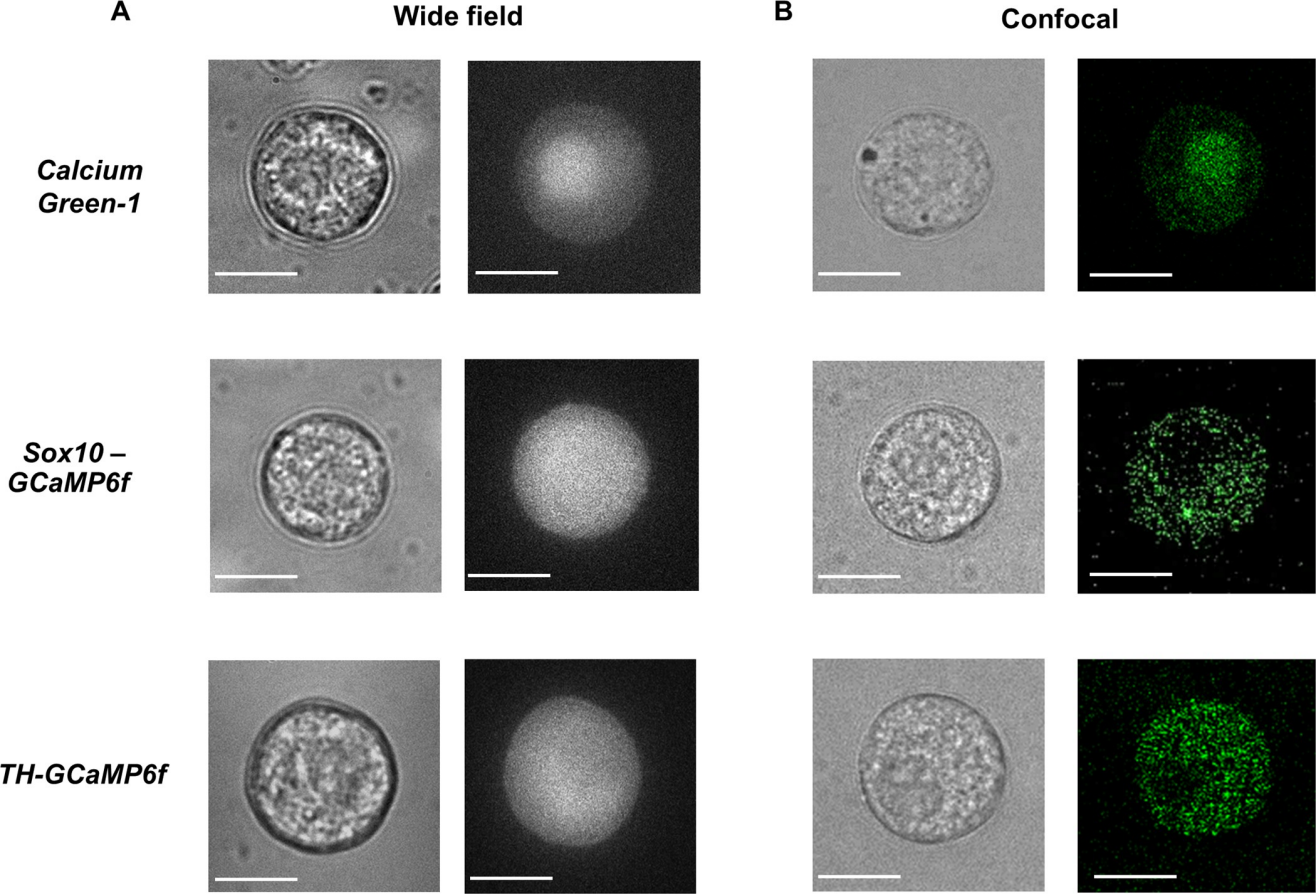
Comparison of brightfield and fluorescence images of Calcium Green-1 loaded and GCaMP6f-expressing ACC. (A) Brightfield and widefield epifluorescence images and (B) brightfield and confocal fluorescence images of *wt* mouse ACC loaded with Calcium Green-1 (top), GCaMP6f-expressing ACC derived from *Sox10-GCaMP6f* mice (middle) and GCaMP6f-expressing ACC derived from *TH-GCaMP6f* mice (bottom). Scale bar = 10 μm.

Fig 2 provides higher magnification brightfield images of ACC in cultures prepared from the adrenal medullae of *wt*, *Sox10-GCaMP6f* and *TH-GCaMP6f* mice. Again, the overall morphology of *wt* and GCaMP6f-expressing cells were found to be comparable. However, whereas the average diameter of cells from *Sox10-GCaMP6f* mice was not statistically different from that of *wt* cells (*Sox10-GCaMP6f* = 15.7 ± 0.2 μm (*n* = 6; *c* = 41) versus *wt* = 14.7 ± 0.1 μm (*n* = 6; *c* = 33), *p* = 0.06)), cells derived from *TH-GCaMP6f* mice tended to be larger (*TH-GCaMP6f* = 17.7 ± 0.3 μm (*n* = 6; *c* = 36)), with the difference being statistically significant (*p* = 0.0001).

#### Basal fluorescence

We next compared baseline fluorescence values of GCaMP6f-expressing ACC in cultures derived from each transgenic mouse line to that of *wt* cells loaded with the Ca^2+^ indicator dye Calcium Green-1, the same chemical Ca^2+^ indicator used in our previous studies of bovine ACC [17,25–27,42]. We employed dye-loading conditions (see Methods) that had been optimized to achieve a visible level of resting fluorescence while also yielding the largest increase in fluorescence in response to a stimulus that triggers a rise in [Ca^2+^]_i_.

Fluorescence images of Calcium Green-1-loaded and GECI-expressing cells are provided in Fig 2. In contrast to dye-loaded *wt* cells, which exhibited fluorescence throughout the cell including high levels in the nucleus, fluorescence in GCaMP6f-expressing cells from each transgenic line was distinctly cytosolic and excluded from the nucleus (Fig 2, middle and bottom rows). In general, the fluorescence intensity in GCaMP6f-expressing ACC was similar between the two types of transgenic mice (Fig 2). However, in some cultures prepared from *TH-GCaMP6f* mice, fluorescence was barely detectable in 30% of the cells. This variability is in addition to the lower percentage of GCaMP6f expression in ACC derived from *TH-GCaMP6f* mice reported above.

#### Spontaneous changes in fluorescence

**Fig 3:**
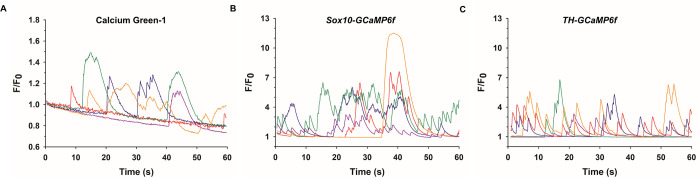
Comparison of spontaneous fluctuations in [Ca^2+^]_i_ in Calcium Green-1 loaded and GCaMP6f-expressing ACC. Representative fluorescence traces of spontaneous Ca^2+^ activity in (A) Calcium Green-loaded ACC, (B) ACC derived from *Sox10-GCaMP6f* mice or (C) from *TH-GCaMP6f* mice. In (A), traces are from ACC in different dishes from the same mouse. In (B) and (C), traces are from ACC from 3 different mice from different litters. In (A), photobleaching is evident by the drop in fluorescence intensity below F/F_0_ = 1.

Cultured ACC from multiple species including cow [26,27,51,52] and mouse [53] exhibit spontaneous elevations of [Ca^2+^]_i_. Such activity, which has also been reported in ACC in *ex vivo* slices of both mouse [53] and rat [54] adrenal gland, is most likely the consequence of spontaneous action potentials generated by Ca^2+^, Na^+^ and K^+^ channels [55–61]. In the present study, we found that 27% of Calcium Green-1 loaded *wt* mouse ACC exhibited transient fluctuations in [Ca^2+^]_i_ that were small in amplitude (F/F_0_ range = 1.2–1.6) and difficult to track over time due to photobleaching (Fig 3A). In contrast, spontaneous Ca^2+^ events, which occurred in a similar percentage (25%) of GCaMP6f-expressing ACC derived from each transgenic line, occurred with considerably higher amplitudes (F/F_0_ range = 2–12), examples of which are shown in Fig 3B and 3C. Note that there was no photobleaching and that the characteristics of the spontaneous events were quite variable, with no regular temporal pattern(s) that could be identified. Such an analysis will be the subject of future studies in order to determine whether specific types of spontaneous activity are sensitive to NEP stimulation, as well as to investigate how spontaneous fluctuations of Ca^2+^ in ACC are related to spontaneous action potentials and contribute to catecholamine release.

### ACC derived from *Sox10-GCaMP6f* mice consistently exhibited the greatest SNR for monitoring physiological stimulus-evoked changes of fluorescence

#### ACC derived from *wt* mice

*In vivo*, stimulation of ACC nAChRs by acetylcholine is the primary event leading to membrane depolarization that causes Ca^2+^ influx via VGCC, culminating in the exocytosis of catecholamines [28,29]. Ca^2+^ imaging studies conducted in cultured bovine ACC loaded with various ratiometric and non-ratiometric fluorescent Ca^2+^ indicator dyes have documented a rapid influx of Ca^2+^ via VGCC following nAChR stimulation [62–64]. Similar studies conducted in cultured mouse ACC also showed that the cells respond to a nAChR agonist by undergoing a rapid rise in [Ca^2+^]_i_ [65,66]. In the present study we used local pressure injection of the nAChR agonist DMPP as a physiologically-relevant strategy to compare Ca^2+^ responses in dye-loaded versus GCaMP6f-expressing mouse ACC. Because in *wt* cultures non-chromaffin cells could comprise as many as 30% of the cells (Table 1) and like ACC would take up the dye, presumptive dye-loaded ACC were identified by reasoning that a cell was indeed an ACC if it responded to DMPP.

**Fig 4:**
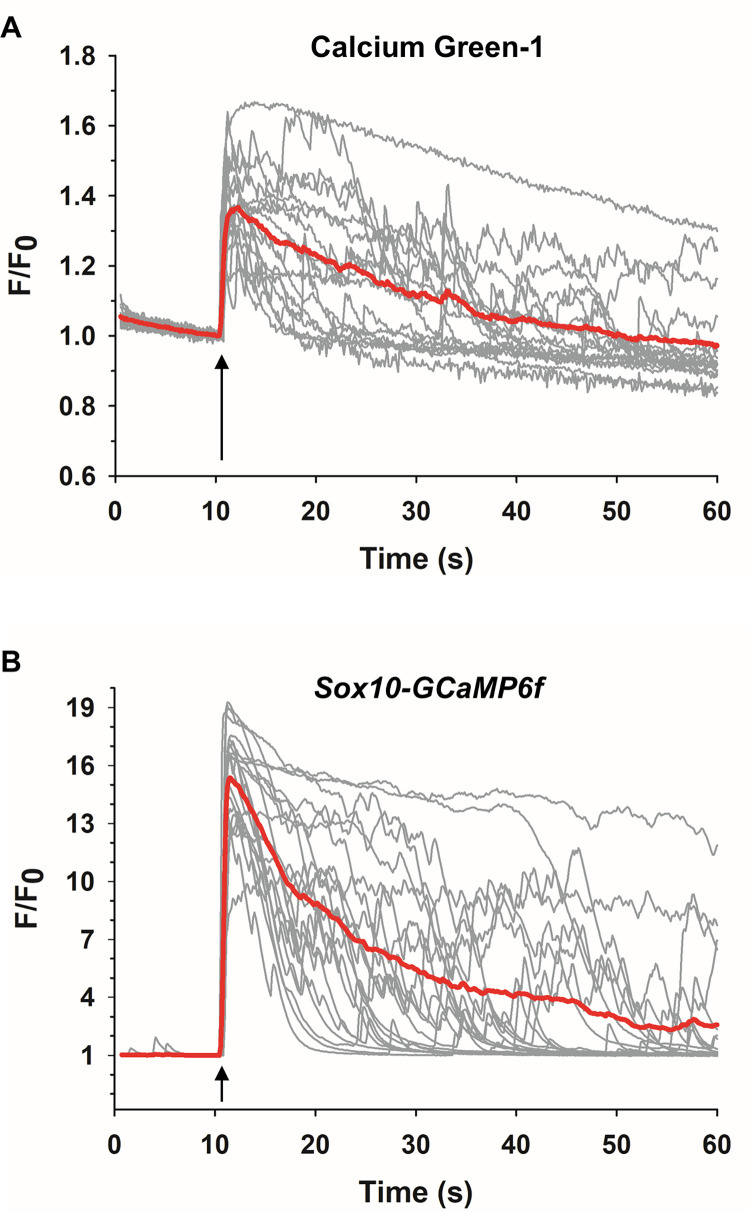
Comparison of Ca^2+^ responses to DMPP in *wt* and GCaMP-expressing ACC derived from *Sox10-GCaMP6f* mice. (A) Ca^2+^ responses to 50 μM DMPP (arrows) in dye-loaded *wt* ACC (*n* = 2, *c* = 17) and (B) in GCaMP6f-expressing ACC (*n* = 3, *c* = 21). Traces show individual cell responses together with the averaged response (red lines). In (A), photobleaching is evident by the drop in fluorescence intensity below F/F_0_ = 1.

Fig 4 shows representative Ca^2+^ responses of Calcium Green-1 loaded *wt* ACC to 50 μM DMPP. The agonist elicited an immediate increase in fluorescence, consistent with previous observations [65,66]. The average time-to-peak of the Ca^2+^ response was 2.6 ± 0.4 s and the fold-change (F/F_0_) was 1.4 ± 0.1. The time course for [Ca^2+^]_i_ to return to baseline was quite variable among cells. In some cells there was a rapid return of fluorescence to basal levels during the monitoring period, with occasional Ca^2+^ spikes occurring at various times after the initial rise in [Ca^2+^]_i_ had returned to baseline (Fig 4A). In other cells, the return of [Ca^2+^]_i_ to baseline was more gradual or else [Ca^2+^]_i_ remained elevated and did not trend toward baseline during the monitoring period (Fig 4A). Overall, the average half-width of the response (i.e., the time interval for [Ca^2+^]_i_ to decline to 50% of the maximal value) was 8.5 ± 1.9 s. Also, in many of the traces photobleaching was evident, which was manifest by a decrease in fluorescence intensity prior to the stimulus and a progressive drop in fluorescence intensity below the initial resting value.

To ensure that the more intense Calcium Green-1 fluorescence signal in the nucleus (Fig 2) did not impact the whole-cell fluorescence measurements, resulting in changes in [Ca^2+^]_i_ that did not reliably reflect those occurring within the cytosol, we compared the amplitude and time course of the changes in [Ca^2+^]_i_ in which the region of interest (ROI) analysis encompassed the whole cell versus an ROI analysis that was restricted to the cytoplasm. We found that fold-change values were similar (whole cell F/F_0_ = 1.8 ± 0.2; cytosolic F/F_0_ = 1.7 ± 0.2; *n* = 2, *c* = 10; *p* = 0.27), indicating that the nuclear presence of the dye observed at basal fluorescence levels did not affect the ability to obtain valid stimulus-evoked fluorescence measurements.

#### ACC derived from *Sox10-GCaMP6f* mice

In contrast to adrenal medullary cultures of *wt* ACC loaded with a Ca^2+^-indicator dye where both ACC and non-ACC would take up the dye and fluoresce, cultures from *Sox10-GCaMP6f* mice had the advantage that ACC could be directly identified since they were the only fluorescent cells in a dish. Fig 4B shows that GCaMP6f-expressing ACC stimulated by 50 μM DMPP exhibited an increase in fluorescence that was as similarly rapid as for *wt* dye-loaded cells (Fig 4A). However, the amplitudes of these fluorescence changes were dramatically higher than those in dye-loaded *wt* cells (*Sox10-GCaMP6f* F/F_0_ = 15.7 ± 0.5 versus *wt* F/F_0_ = 1.4 ± 0.1). The responses occurred with a slightly faster rise time (time-to-peak = 1.1 ± 0.1 s) than for *wt* dye-loaded cells (time-to-peak = 2.6 ± 0.4 s) that was statistically significant (*p*<0.001) whereas the half-width (10.8 ± 1.9 s versus 8.5 ± 1.9 s for *wt*) was similar (*p* = 0.403). As observed in dye-loaded cells, post-stimulus Ca^2+^ spikes were present but were more pronounced in amplitude. Also, responses were triggered regardless of whether the cells exhibited spontaneous activity or were quiescent. Finally, GCaMP6f-expressing cells failed to exhibit any evidence of photobleaching as found for dye-loaded cells. From these findings we conclude that the high SNR and thus the enhanced monitoring of stimulus-evoked Ca^2+^ responses that can be achieved in GCaMP6f-expressing ACC relative to *wt* cells loaded with a Ca^2+^ indicator dye occurred without any apparent adverse effect on cellular Ca^2+^ handling.

#### ACC derived from *TH-GCaMP6f* mice

**Fig 5:**
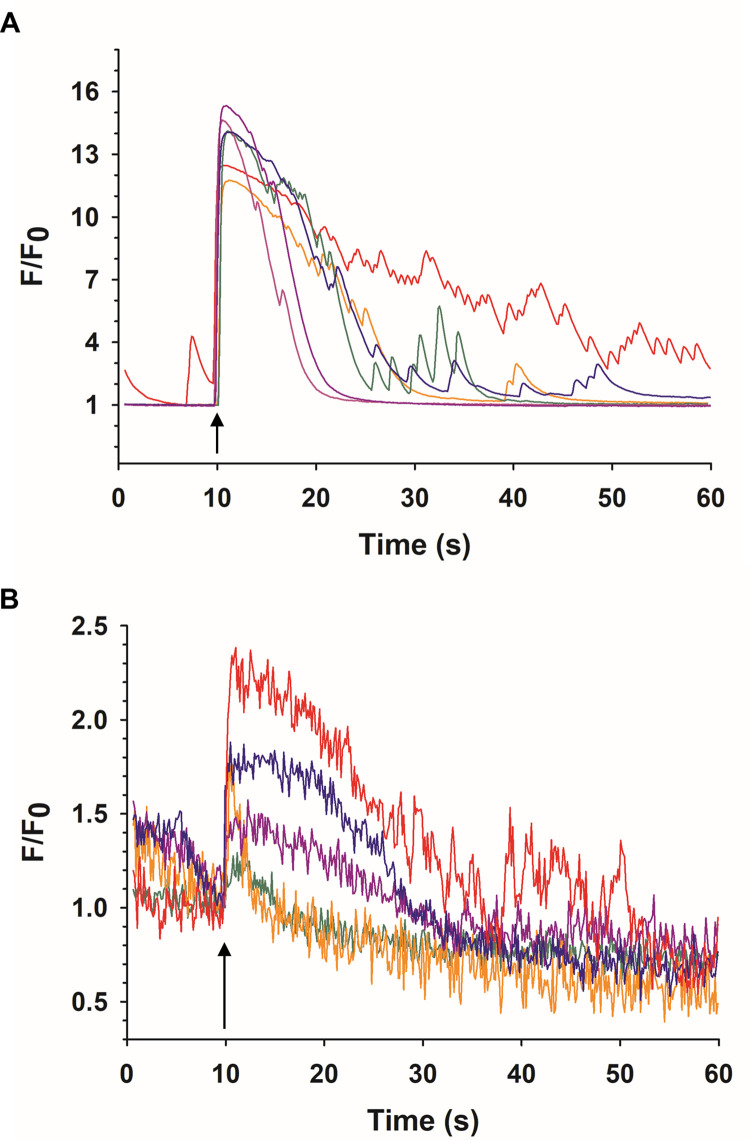
Effect of different GCaMP6f expression levels on DMPP-induced Ca^2+^ responses in *TH-GCaMP6f*-expressing ACC. Representative fluorescence traces of Ca^2+^ responses evoked by 50 μM DMPP (arrows) in cells exhibiting (A) a “high” (*n* = 3, *c* = 13) or (B) “low” (*n* = 3, *c* = 8) level of GCaMP6f expression. In (B), photobleaching is evident by the drop in fluorescence intensity below F/F_0_ = 1.

A parallel study carried out to assess nAChR-evoked Ca^2+^ responses in ACC derived from *TH-GCaMP6f* mice revealed that Ca^2+^ responses varied in a manner that correlated with the expression level of GCaMP6f fluorescence. For example, ACC from *TH-GCaMP6f* mice whose basal fluorescence level was similar to that in cells from *Sox10-GCaM6f* mice (Fig 2), which we refer to as “high”, exhibited changes in fluorescence in response to application of 50 μM DMPP (Fig 5A) that also were similar in amplitude to those derived from *Sox10-GCaMP6f* mice *(TH-GCaMP6f* “high” F/F_0_ = 14.7 ± 0.8). Thus, the majority of cells exhibited high SNR responses to nAChR stimulation. In ACC from *TH-GCaMP6f* mice whose basal fluorescence levels were barely detectable, which we refer to as “low”, the response to DMPP resembled that observed in dye-loaded *wt* cells (*TH-GCaMP6f* “low” F/F_0_ = 1.9 ± 0.2), exhibiting a poor SNR (Fig 5B). In addition, significant photobleaching was present and the fluorescence traces appeared noisy. We currently have no explanation for this phenomenon.

Table 2 summarizes the characteristics of nAChR-evoked Ca^2+^ responses in Calcium Green-1 loaded *wt* ACC and in the two different GCaMP6f-expressing ACC. First, the kinetic characteristics of nAChR-evoked changes in [Ca^2+^]_i_ were similar in *wt* cells and in each type of GCaMP6f-expressing cell, indicating that the genetic targeting of GCaMP6f to mouse ACC does not alter the Ca^2+^ response to the nAChR stimulus. Second, relative to the fluorescent Ca^2+^ indicator dye, cGaMP6f greatly enhanced the SNR for visualizing and recording changes in [Ca^2+^]_i_ in the absence of photobleaching. Thus, while the kinetics of physiological stimulus-driven Ca^2+^ responses in mouse ACC are similar between dye-loaded versus GECI-expressing ACC, the lack of photobleaching and enhanced SNR favor the use of ACC expressing GCaMP6f as a tool to probe responses to Ca^2+^-evoking stimuli. Third, an enhanced SNR for visualizing and recording changes in [Ca^2+^]_i_ was found consistently in ACC derived from *Sox10-GCaMP6f* mice but not in ACC derived from *TH-GCaMP6f* mice. Therefore, because GCaMP6f expression specifically in catecholaminergic cells was more scattered and with more variable basal fluorescence intensity between cells than GCaMP6f expression in neural crest derived cells, we chose to restrict our studies of NEP stimulation to ACC derived from *Sox10-GCaMP6f* mice.

**Table 2:** Comparison of DMPP-evoked Ca^2+^ responses in ACC derived from *wt*, *Sox10-GCaMP6f* and *TH-GCaMP6f* mice.

	F/F_0_	TTP^a^	HW^b^
		(s)	(s)
***wt***	1.4 ± 0.1	2.6 ± 0.4	8.5 ± 1.9
***Sox10-GCaMP6f***	15.7 ± 0.5***	1.1 ± 0.1***	10.8 ± 1.9
***TH-GCaMP6f***			
**“high" F**	14.7 ± 0.8***	1.2 ± 0.1**	12.1 ± 0.9
**“lo” F**	1.9 ± 0.2*	1.1 ± 0.1*	12.3 ± 2.9

^a^ response time-to-peak.

^b^ response half-width.

*p<0.05

**p<0.005

***p<0.0001 relative to *wt*.

### Ca^2+^ responses evoked by a 5 ns pulse in ACC derived from *Sox10-GCaMP6* mice exhibited a high SNR relative to dye-loaded *wt* cells

#### Ca^2+^ response kinetics

Our next goal was to test whether 5 ns electric pulses evoked Ca^2+^ responses in murine ACC similar to those reported for bovine ACC [25–27,42]. For this determination, *wt* murine ACC were loaded with Calcium Green-1 and exposed to a single 5 ns pulse using the same experimental approach and pulse exposure setup as in previous studies of bovine ACC [42,43]. Cells were first exposed to 50 μM DMPP to ensure that target cells were indeed ACC. Once an ACC was identified in this manner, the same cell was exposed to a 5 ns pulse two minutes later.

In response to a pulse with an electric field amplitude of 5 MV/m, which for bovine ACC is the lowest field amplitude at which all cells are stimulated [42], only 30% of murine ACC exhibited a rise in [Ca^2+^]_i_. When a cell did not respond to the pulse, applying a second pulse at 8 MV/m or higher always elicited a rise in [Ca^2+^]_i_, suggesting that unlike bovine ACC, murine ACC required an electric field of at least 8 MV/m to always evoke a Ca^2+^ response. Based on these results, pulses were applied at 8 MV/m in subsequent experiments.

**Fig 6:**
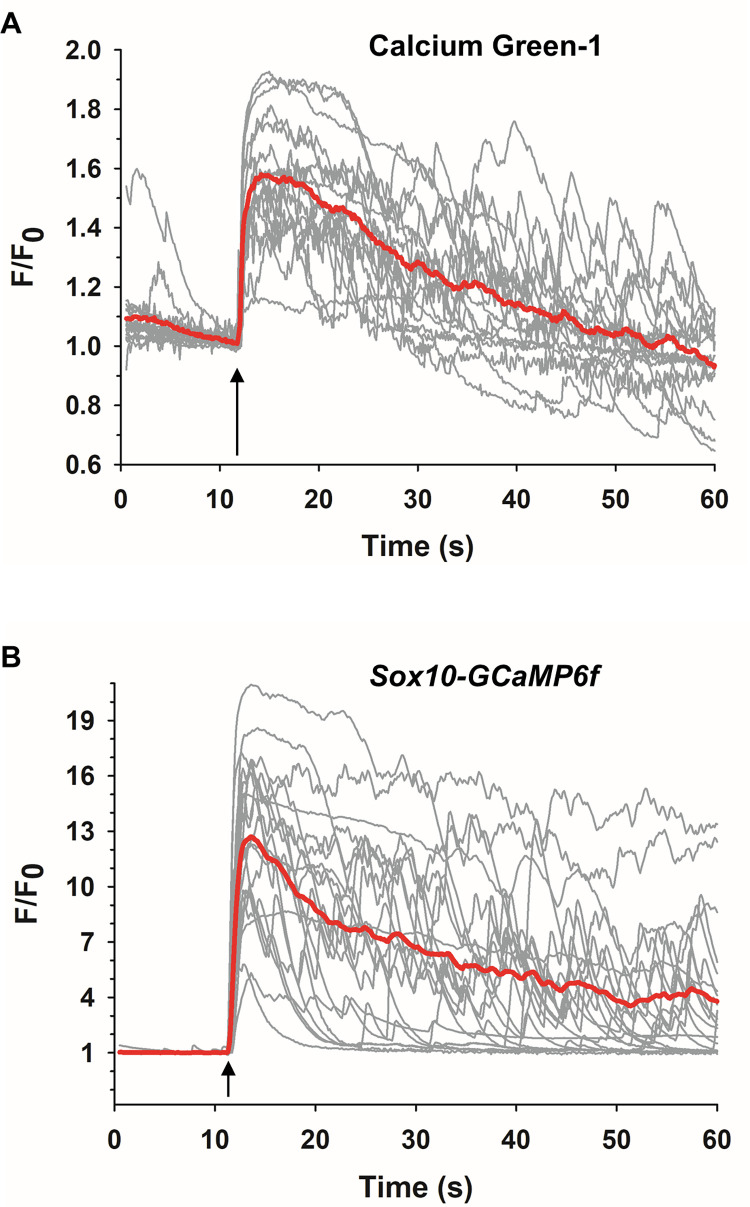
Comparison of Ca^2+^ responses in *wt* and GCaMP-expressing mouse ACC exposed to a 5 ns pulse. (A) Ca^2+^ responses evoked by a 5 ns, 8 MV/m pulse (arrows) in *wt* dye-loaded ACC (*n* = 3, *c* = 16) and (B) in GCaMP6f-expressing ACC (*n* = 3, *c* = 20). Traces show individual cell responses together with the averaged response (red lines). In (A), photobleaching is evident by the drop in fluorescence intensity below F/F_0_ = 1.

Fig 6A shows representative Ca^2+^ traces for dye-loaded *wt* ACC exposed to a single 5 ns, 8 MV/m pulse. The pulse caused an average fluorescence increase of 1.6 ± 0.1, a fold-change comparable to that observed in response to a 5 ns pulse in bovine ACC [25–27,42], demonstrating that the NEP elicits a similar Ca^2+^ response across species. The response time-to-peak was 2.9 s ± 0.3 s and the half-width was 13.5 ± 1.5 s, with post-stimulus Ca^2+^ spikes similar to those seen in cells stimulated with DMPP occurring in many cells. Photobleaching was evident by the decay of the fluorescence signal pre- and post-stimulus.

Similar to *wt* dye-loaded cells, a Ca^2+^ response could be elicited in some (30%) but not all GCaMP6f-expressing ACC with pulses whose electric field was 5 MV/m and in all ACC when the pulse was applied at 8 MV/m. Thus, dye-loaded *wt* and GCaMP6f-expressing mouse ACC displayed similar sensitivity to a 5 ns pulse, indicating that the genetic manipulation of the cells did not alter their responsiveness to the NEP stimulus.

Like DMPP-evoked Ca^2+^ responses in GCaMP6f-expressing ACC, NEP-evoked Ca^2+^ responses in these cells (Fig 6B) were higher in amplitude (F/F_0_ = 13.7 ± 0.9) than those of dye-loaded *wt* ACC while the response time-to-peak and half-width durations were similar (time-to-peak = 2.5 s ± 0.3 s; half-width = 13.1 ± 1.8 s). These results are summarized in Table 3. Given this demonstration that the SNR of the response was so much higher in GECI-expressing than in dye-loaded ACC without altering the kinetic characteristics of the Ca^2+^ response, we continued to examine the effects of NEP in GCaMP6f-expressing cells only.

**Table 3:** Comparison of 5 ns—evoked Ca^2+^ responses in ACC derived from *wt* and *Sox10-GCaMP6f* mice.

	F/F_0_	TTP^a^	HW^b^
		(s)	(s)
***wt***	1.6 ± 0.1	2.9 ± 0.3	13.5 ± 1.5
***Sox10-GCaMP6f***	13.7 ± 0.9***	2.5 ± 0.3	13.1 ± 1.8

^a^ response time-to-peak.

^b^ response half-width.

***p < 0.0001.

#### Requirement for extracellular Ca^2+^

**Fig 7:**
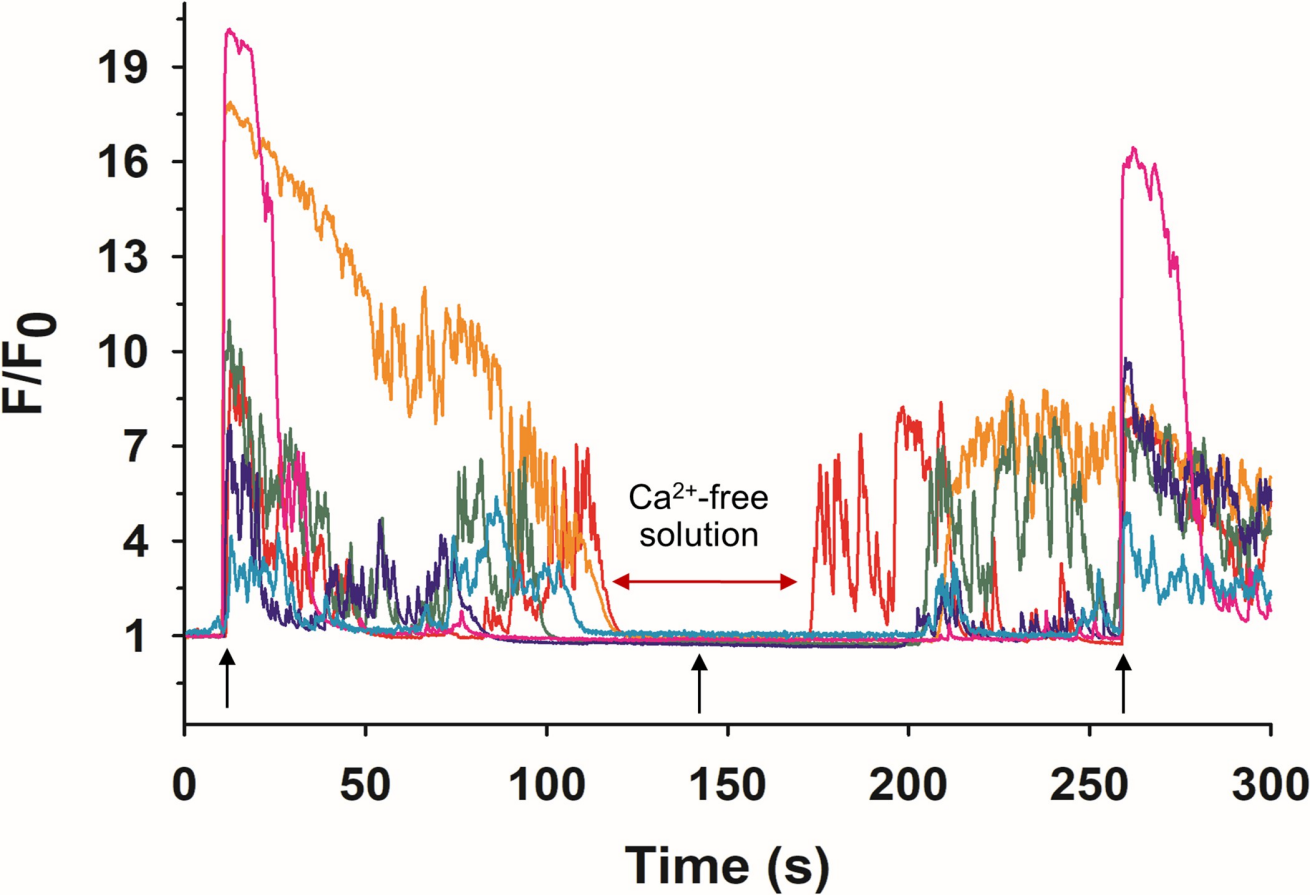
Effect of eliminating extracellular Ca^2+^ on 5 ns-elicited Ca^2+^ responses in GCaMP6f-expressing ACC. Cells attached to fibronectin-coated glass coverslips were placed in a perfusion chamber. Cells were continuously perfused at a rate of 1 ml/min, first with Ca^2+^-containing BSS, followed by Ca^2+^-free BSS, and again with Ca^2+^-containing BSS. Arrows indicate each time a 5 ns, 8 MV/m pulse was applied to the cells (*n* = 3, *c* = 11).

In bovine ACC, a 5 ns pulse evokes a rise in [Ca^2+^]_i_ that is the result of Ca^2+^ entering the cells and not release of Ca^2+^ from internal stores due to electropermeabilization of the membranes of Ca^2+^-storing organelles [25,42,43], as has been reported for a variety of other cell types that include both excitable [67,68] and non-excitable [69,70] cells. In the present study we used the same approach to make this determination, namely to expose cells to a 5 ns pulse in Ca^2+^-free BSS. As shown in Fig 7, a NEP failed to elicit a Ca^2+^ response in GCaMP6f-expressing mouse ACC in Ca^2+^-free BSS. When Ca^2+^ was reintroduced, the cells were again able to elicit such a response to the 5 ns electric stimulus. Thus, similar to that of bovine ACC, the Ca^2+^ response of murine ACC to a 5 ns pulse is mediated by Ca^2+^ influx. An additional point is that in each of these recordings, spontaneous Ca^2+^ activity also disappeared in the absence of bath Ca^2+^ and reappeared with the reintroduction of Ca^2+^ (Fig 7). This finding supports the notion that these Ca^2+^ events are likely the consequence of spontaneous action potentials that lead to Ca^2+^ influx via VGCC.

#### Role of VGCC

**Fig 8:**
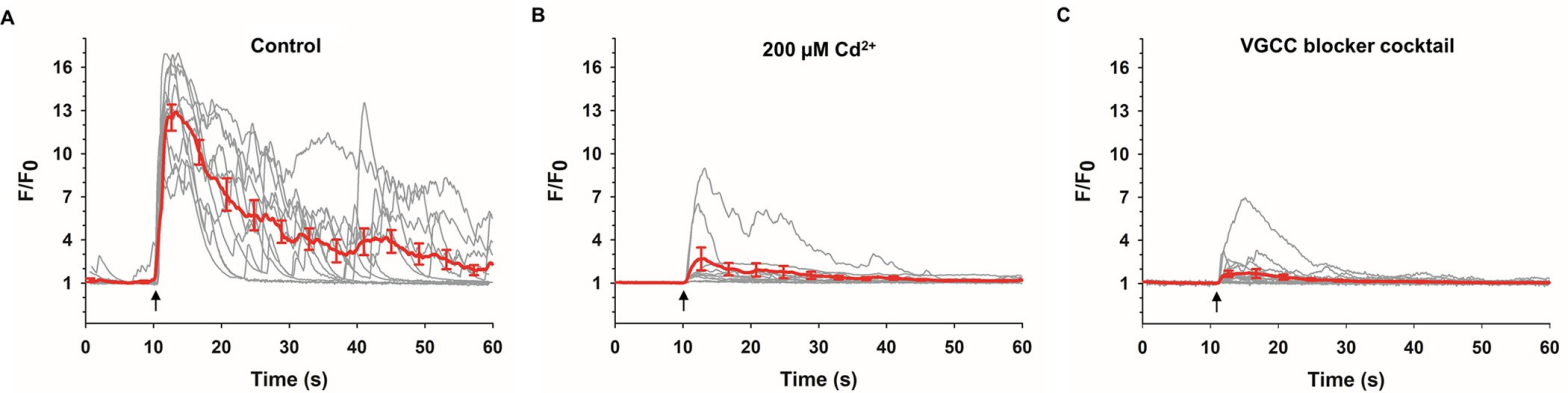
Effect of blocking VGCC on Ca^2+^ responses evoked by a 5 ns pulse. Individual cell responses to a 5 ns, 8 MV/m pulse (arrow), together with the averaged response ± SEM (red line), in the absence (A; *n* = 4, *c* = 13) or presence of 200 μM Cd^2+^ (B; *n* = 2, *c* = 10) or presence of a cocktail of VGCC blockers consisting of 3 μM ω-CTX GVIA, 2 μM ω-Aga IVA, 1 μM SNX-482, 3 μM nifedipine (C; *n* = 2, *c* = 15).

In order to determine if NEP-induced Ca^2+^ influx was mediated by VGCC, as is the case with bovine ACC [25–27], murine GCaMP6f-expressing ACC were exposed to a 5 ns pulse in BSS containing Cd^2+^, an inorganic, non-selective blocker of VGCC. As shown in Fig 8A and 8B, the rise in [Ca^2+^]_i_ was essentially absent in the majority of cells and when present, the amplitude of the responses was significantly attenuated relative to control cells where F/F_0_ = 12.9 ± 0.9 (time-to-peak = 1.7 ± 0.1 s; half-width = 9.8 ± 0.2 s). We next exposed cells to a 5 ns pulse in the presence of blockers that are specific for the types of VGCC expressed in cultured mouse ACC, including P/Q type (Ca_v_2.1), N type (Ca_v_2.2) and L type (Ca_v_1.2 and 1.3) channels, all of which are expressed in cultured bovine ACC, as well as R type (Ca_v_2.3) channels, which are expressed in murine but not bovine ACC (reviewed in [71]). To simultaneously block all these VGCC, we used a cocktail that included ω-conotoxin GVIA3 (3 μM), ω-agatoxin IVA (2 μM), SNX-482 (1 μM), and nifedipine (3 μM) to inhibit, respectively, N type, P/Q type, R type and L type channels. The presence of functional T-type (Ca_v_3.2) channels in ACC derived from non-stressed adult mice is unresolved [72]; hence these channels were not considered in this study. As shown in Fig 8C, the majority of cells pretreated with the VGCC blocker cocktail did not undergo a rise in [Ca^2+^]_i_, similar to what has been found for bovine ACC treated with a cocktail of VGCC antagonists [27]. In those cells that exhibited an increase in Ca^2+^, the amplitude of the responses was significantly attenuated, as found for Cd^2+^. In the presence of either Cd^2+^ or the VGCC blocker cocktail, spontaneous Ca^2+^ activity was never observed, similar to cells in Ca^2+^-free BSS. Taken together, these results show that in GCAMP6f-expressing murine ACC, as for bovine ACC, VGCC are mainly responsible for mediating the rise in [Ca^2+^]_i_ induced by a 5 ns pulse.

**Fig 9:**
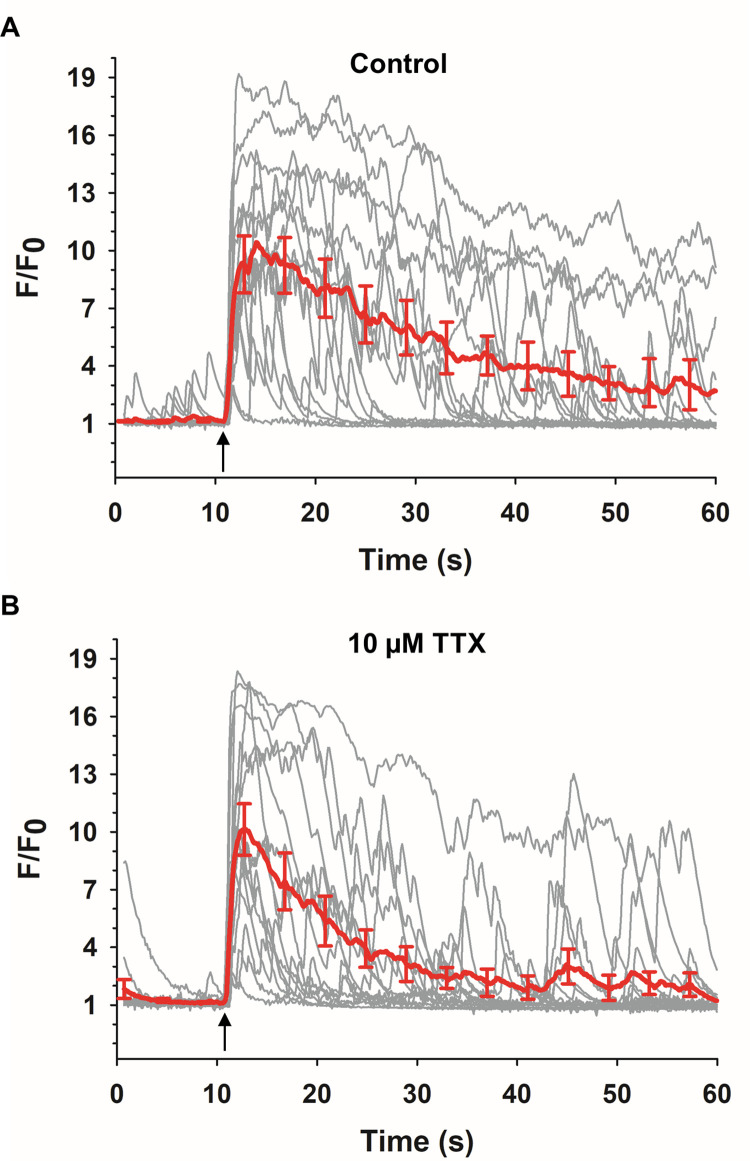
Effect of blocking VGSC with TTX on Ca^2+^ responses evoked by a 5 ns pulse. Individual cell responses to a 5 ns pulse together with the averaged response ± SEM (red line), in mouse GCaMP6f-expressing ACC, in the absence (A; *n* = 3, *c* = 15) or presence (B; *n* = 3, *c* = 15) of 10 μM tetrodotoxin (TTX). Cells in (A) and (B) are from the same 3 mice. Arrows indicate the times at which a 5 ns, 8 MV/m pulse was applied to the cells.

We are still investigating the membrane-depolarizing mechanism by which VGCC are activated in bovine ACC exposed to a 5 ns pulse. As we reported in Yang et al. [73], and also as mentioned in the review by Pakhomov and Pakhomova [8], a possible mechanism may involve activation of a yet-to-be identified Na^+^-conducting ion channel. However, what has been established is that tetrodotoxin- (TTX-) sensitive voltage-gated Na^+^ channels (VGSC) do not play a role [27]. Since mouse ACC reportedly express TTX-sensitive Na_v_1.7 and Na_v_1.3 VGSC [74], we tested whether a 5 ns pulse could elicit a Ca^2+^ response in GCAMP6f-expressing mouse ACC in the presence of this drug (Fig 9). We found that the NEP-evoked Ca^2+^ response time-to-peak (1.9 ± 0.1 s versus 1.7 ± 0.1s, control versus TTX, respectively; *p* = 0.168) and response amplitude (F/F_0_ = 10.6 ± 0.9 versus 10.5 ± 0.7, control versus TTX, respectively; *p* = 0.931), were similar in the absence or presence of TTX, suggesting that like bovine ACC, NEP activation of VGCC in murine ACC occurs independently of VGSC. There was, however, a difference in half-width (18.9 ± 1.5 s versus 10.3 ± 1.0 s, control versus TTX, respectively) that was statistically significant (*p* = 0.0001).

#### NEP exposure does not impact subsequent responses to DMPP

**Fig 10:**
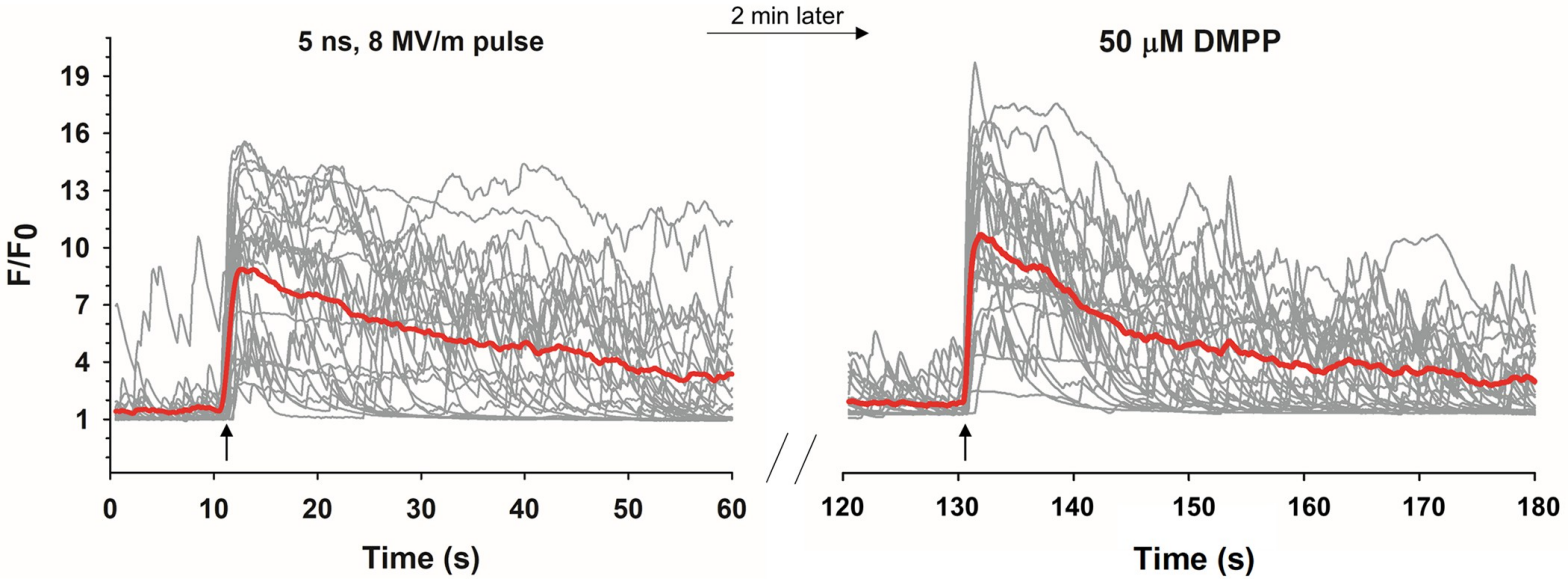
nAChR-evoked Ca^2+^ responses after NEP exposure. Representative fluorescence traces together with the averaged responses (red line) showing the Ca^2+^ response in mouse GCaMP6f-expressing ACC to a 5 ns pulse and the response of the same cells to DMPP 2 min later (*n* = 3, *c* = 26). The arrow indicates when the stimulus was applied.

To further demonstrate the advantages provided by the high SNR and photostability of the GECI for investigating the effects of NEP exposure on ACC, we exposed GCAMP6f-expressing mouse ACC first to a 5 ns, 8 MV/m pulse and 2 min later stimulated the cells with DMPP when [Ca^2+^]_i_ had returned to baseline. As shown in Fig 10, DMPP elicited a robust Ca^2+^ response with similar characteristics as those in cells not previously exposed to a NEP (Fig 4). Additionally, spontaneous Ca^2+^ activity was observed post-pulse, prior to DMPP, just as found before the pulse. Thus, stimulation of mouse GCaMP6f-expressing ACC with a NEP does not inhibit their ability to respond to physiological stimulation or to exhibit spontaneous activity, thereby supporting the notion that these pulses do not cause damage to these cells. These results also provide further evidence of 5 ns-evoked ACC stimulation that mimics nAChR-mediated ACC stimulation since the Ca^2+^ responses were similar.

## Conclusion

Fluorescence Ca^2+^ imaging is a valuable research tool that has allowed for real-time monitoring of stimulus-evoked Ca^2+^ responses in a variety of cultured cells *in vitro*. With the goal of advancing the study of NEP-elicited Ca^2+^ dynamics in neural-type ACC, we developed a genetic strategy that provides a high SNR for monitoring stimulus-evoked Ca^2+^ transients as well as spontaneous Ca^2+^ events. To our knowledge, this is the first report demonstrating the suitability of using the GECI GCaMP6f to monitor [Ca^2+^]_i_ in mouse ACC derived from transgenic mice. The strategy we describe not only yields a more sensitive method to image Ca^2+^ responses in isolated ACC but also provides a basis upon which to investigate Ca^2+^ responses in ACC in intact tissue. Thus, chromaffin cell biologists have a new reporter system to more precisely explore Ca^2+^ activity in these neuroendocrine cells both *in vitro* and *in situ*. For us, this means the ability to establish whether the parameters of NEP exposure that achieve optimal activation of ACC in culture differ from those that activate them in the more complex and physiologically relevant environment of intact tissue. A study of this kind has not yet been undertaken for any cell type and for ACC would represent an essential first step to obtain fundamental information regarding the potential use of NEP to stimulate neurosecretion *in vivo*, including from the adrenal gland itself [75]. Validation of the utility and performance of GCaMP6f in ACC to investigate the nature of spontaneous Ca^2+^ events as well as stimulus-evoked changes in [Ca^2+^]_i_ *in situ* (i.e., in thin adrenal gland slices) is currently underway.

Despite reports that transgenic expression of GECI can exert undesirable effects, such as interference with the gating and signaling of L-type VGCC by GCaMP [76], we did not detect any difference in Ca^2+^ response kinetics between *wt* and GCaMP6f-expressing murine ACC, suggesting that the molecular pathways underlying this response (e.g., VGCC) were unaffected in ACC expressing GCaMP6f. Nevertheless, as our studies progress into more functional studies of ACC activation (e.g., catecholamine release), we will monitor for these and other potential deleterious effects of GCaMP6f expression, one being reduced synaptic vesicle release probability [77].

## Supporting information

S1 Fig Immunohistochemical expression of GCaMP6f in adrenal gland of *Sox10-GCaMP6f* and *TH-GCaMP6f* mice. Adrenal gland cross sections *Sox10-GCaMP6f* mice (upper row) or TH-*GCaMP6f* mice (lower row) were stained with antibodies against GFP to mark GCaMP6f-expressing cells (left column, green) and TH to mark ACC (right column, red). Note the greater correlation between GFP and TH in *Sox10-GCaMP6f* versus *TH-GCaMP6f* mice, which results from greater recombination efficiency of GCaMP6f expression in ACC in *Sox10-GCaMP6f* mice.
(TIF)

S2 Fig Immunohistochemical detection of SGC in mouse adrenal gland. Adrenal gland cross sections from *wt* mice were stained with antibodies against TH to mark ACC and S100 to mark SGC. Low-magnification images (left column) demonstrate that immunoreactivity for each of these cell-specific markers is observed in the adrenal medulla (M) but not cortex (C). High-magnification images (right two columns) show distinct immunohistochemical staining patterns for each of these markers as well as nuclear counterstaining with Hoechst 33342.
(TIF)
