# Supplementary figures and images for: High signal-to-noise imaging of spontaneous and 5 ns electric pulse-evoked Ca2+ signals in GCaMP6f-expressing adrenal chromaffin cells isolated from transgenic mice
Source: PLoS One. 2023 Mar 31;18(3):e0283736. doi: 10.1371/journal.pone.0283736 (PMC10065239; doi:10.1371/journal.pone.0283736)

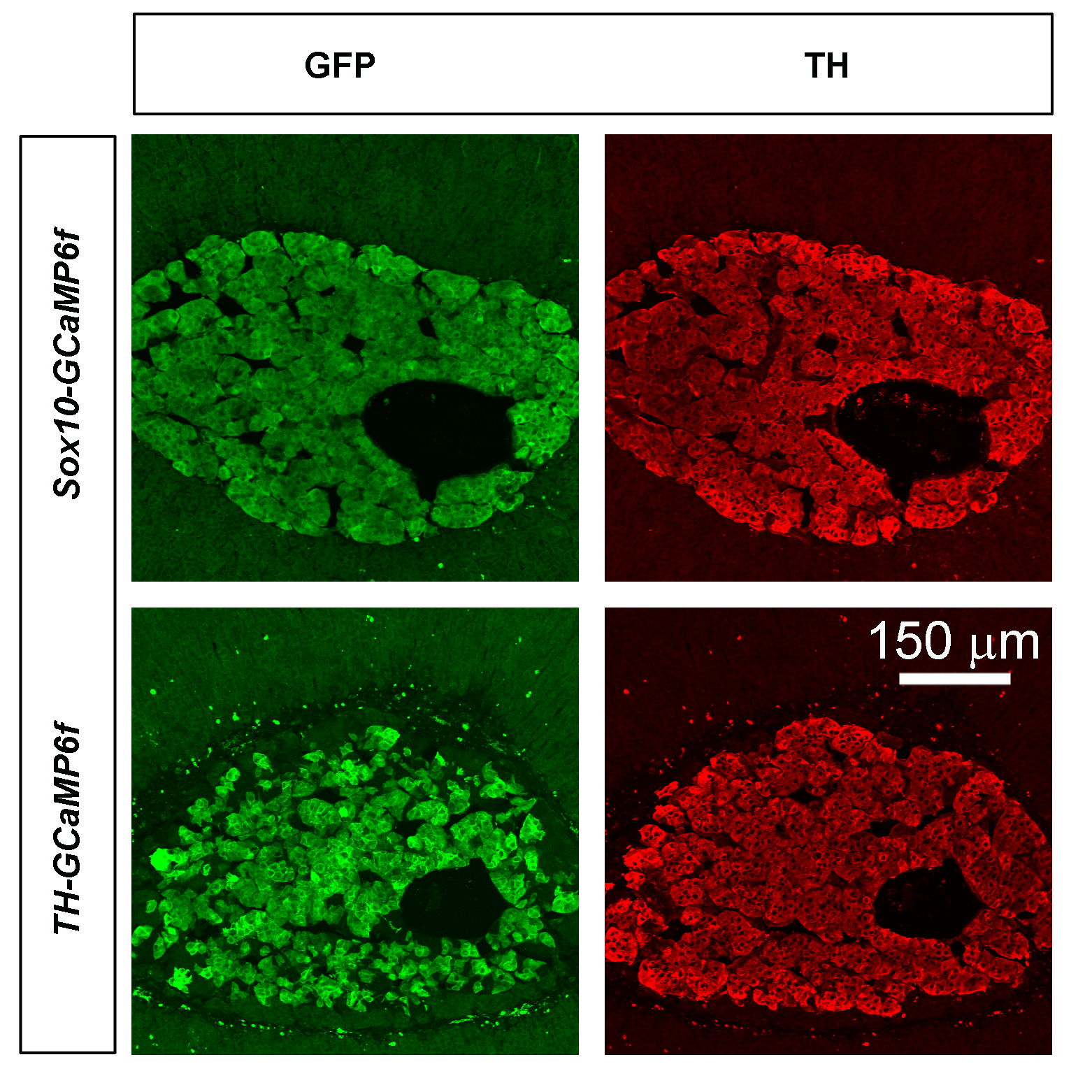

Supplement: S1 Fig — Adrenal gland cross sections Sox10-GCaMP6f mice (upper row) or TH-GCaMP6f mice (lower row) were stained with antibodies against GFP to mark GCaMP6f-expressing cells (left column, green) and TH to mark ACC (right column, red). Note the greater correlation between GFP and TH in Sox10-GCaMP6f versus TH-GCaMP6f mice, which results from greater recombination efficiency of GCaMP6f expression in ACC in Sox10-GCaMP6f mice. (TIF) [file pone.0283736.s001.tif]

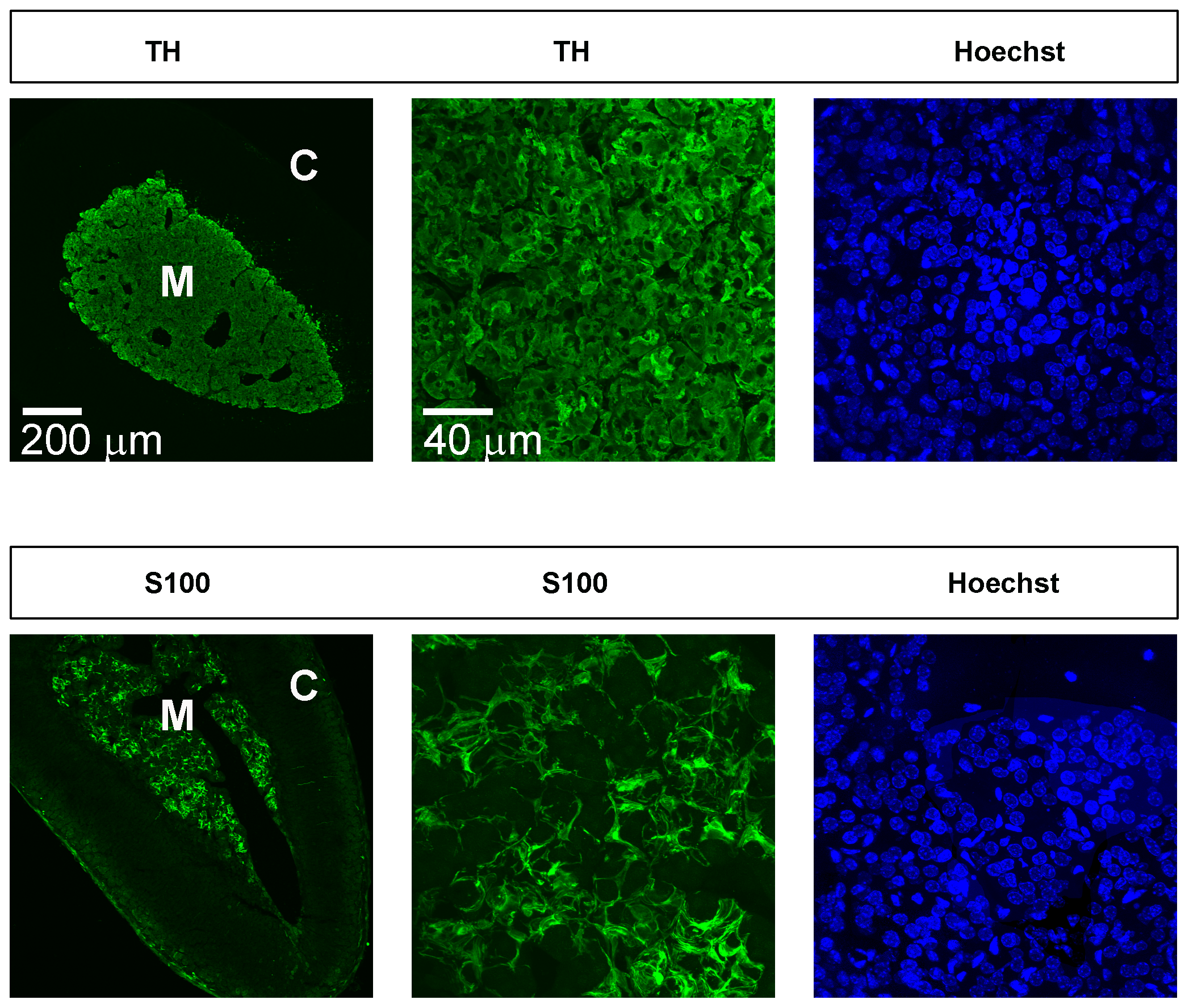

Supplement: S2 Fig — Adrenal gland cross sections from wt mice were stained with antibodies against TH to mark ACC and S100 to mark SGC. Low-magnification images (left column) demonstrate that immunoreactivity for each of these cell-specific markers is observed in the adrenal medulla (M) but not cortex (C). High-magnification images (right two columns) show distinct immunohistochemical staining patterns for each of these markers as well as nuclear counterstaining with Hoechst 33342. (TIF) [file pone.0283736.s002.tif]
